# Compressed collagen and decellularized tissue – novel components in a pipeline approach for the study of cancer metastasis

**DOI:** 10.1186/s12885-018-4533-0

**Published:** 2018-06-01

**Authors:** Shirley Jean Keeton, Jean Marie Delalande, Mark Cranfield, Alan Burns, Philip Richard Dash

**Affiliations:** 1 Cell Migration Lab, School of Biological Sciences, University of Reading, Reading, RG6 6UB UK; 2 Blizard Institute, Barts & The London School of Medicine & Dentistry, The Blizard Building, Centre for Immunology, Queen Mary, University of London, 4 Newark Street, London, E1 2AT UK; 3 Natural Biosciences SA, Seestrasse 155A, Kilchberg, 8802 Zurich, Switzerland; 4 Institute of Child Health, University College London, London, WC1E 6BT UK

**Keywords:** Metastasis, CAM, 3D culture, Decellularization, 3D model, Extracellular matrix

## Abstract

**Background:**

Metastasis is a complex process which is difficult to study and model. Experimental ingenuity is therefore essential when seeking to elucidate the biological mechanisms involved.

Typically, in vitro models of metastasis have been overly simplistic, lacking the characteristic elements of the tumour microenvironment, whereas in vivo models are expensive, requiring specialist resources. Here we propose a pipeline approach for the study of cell migration and colonization, two critical steps in the metastatic cascade.

**Methods:**

We used a range of extracellular matrix derived contexts to facilitate a progressive approach to the observation and quantification of cell behaviour in 2D, 3D and at border zones between dimensions. At the simplest level, cells were set onto collagen-coated plastic or encapsulated within a collagen matrix. To enhance this, a collagen compression technique provided a stiffened, denser substrate which could be used as a 2D surface or to encapsulate cells. Decellularized tissue from the chorioallantoic membrane of the developing chicken embryo was used to provide a more structured, biologically relevant extracellular matrix-based context in which cell behaviour could then be compared with its in vivo counterpart.

**Results:**

Cell behaviour could be observed and quantified within each context using standard laboratory techniques of microscopy and immunostaining, affording the opportunity for comparison and contrast of behaviour across the whole range of contexts. In particular, the temporal constraints of the in vivo CAM were removed when cells were cultured on the decellularized CAM, allowing for much longer-term cell colonization and cell-cell interaction.

**Conclusions:**

Together the assays within this pipeline provide the opportunity for the study of cell behaviour in a replicable way across multiple environments. The assays can be set up and analysed using easily available resources and standard laboratory equipment. We believe this offers the potential for the detailed study of cell migration and colonization of tissue, essential steps in the metastatic cascade. Also, we propose that the pipeline could be used in the wider arena of cell culture in general with the increasingly more complex contexts allowing cell behaviours and interactions to be explored in a stepwise fashion in an integrated way.

## Background

Metastasis is the leading cause of cancer-related death and has as such been an important area of investigation into the mechanisms and processes involved. Metastasis is, however, a complex, multi-stage process which due to its temporal and unpredictable nature is difficult to study. The development of suitable models to elucidate the mechanisms and processes involved has been challenging due to the inherent nature of the process.

The outward spread of cancer from a tumour occurs in several stages: cellular escape, invasion, intravasation, extravasation, seeding and colonization at distant sites [1, 2]. Both in vitro and in vivo experimental models have been used to gain further insight into metastatic mechanisms. Until recently in the main, in vitro models have been relatively simple, using extracellular matrix (ECM) components and tissue culture approaches to investigate the escape and migration of cells [3–5]. With a growing emphasis on the importance of the tumour microenvironment, it has become clear that both structural and cellular components of the tissue architecture play a crucial role in the metastatic process [6–8]. Models of metastasis, therefore, need to reflect the complexity of the tumour microenvironment, the conduits involved in the metastatic processes and the tissue architecture and features of sites of metastatic seeding and colonization. Better models will enable not only a more detailed understanding of the processes involved but also provide improved opportunities for the testing of candidate molecules before drugs trials.

Advances in the manufacture and use of biomaterials in the biomedical field have led to a range of materials and approaches that are potentially available for the culture of cells in more relevant biological contexts [9–11]. The development of tissue engineering approaches using patient-derived material now also provides the opportunity to generate more natural and complex materials as substrates for 3D cell culture and the study of disease.

Based on a biomaterials approach, here we propose a set of in vitro assays of increasing complexity which were used in comparison with a well characterized in vivo assay, the chicken chorioallantoic membrane (CAM) assay, for the study of cell migration and colonization in a 3D tissue context [12–14].

## Methods

### Cell culture

HT1080 human fibrosarcoma cells, MCF-7 human breast cancer cells, MDA-MB-231 human breast cancer cells and SK-MEL-28 human melanoma cells (HPA ECCC) were routinely cultured and passaged in Greiner Bio-one flasks placed in a humidified incubator at 37 °C/ 5% CO_2_, in DMEM (low glucose with glutamine) supplemented with 10% Fetal Bovine Serum and 1% Penicillin/Streptomycin (Gibco).

### 3D culture

Rat Tail Type I Collagen (BD Bioscience) was used for the construction of collagen gels, diluted and adjusted to pH 7.5 according to the manufacturer’s instructions. Simple collagen gels were set onto tissue culture plastic with cells seeded either over the surface or encapsulated within. Where fibronectin was incorporated into the collagen gel, human recombinant fibronectin was added to the collagen mix at a final concentration of 10 μg/ml. Compressed collagen discs were prepared using collagen at 2 mg/ml set in a 24-well culture dish then compressed between two fine nylon mesh layers bounded by layers of filter paper and glass plates then weighted to 126 g for 2.5 or 5 min [15]. Compressed collagen discs were either left to free-float bathed in medium or set into a 1 mg/ml collagen gel. Where cells were encapsulated into compressed collagen, a copper grid (1.7 mm hole-size) was used above the nylon mesh layer to avoid cells being crushed during the compression step.

### Chick Chorioallantoic membrane (CAM) assay

The chicken egg chorioallantoic membrane is highly vascularized, comprising three layers which together provide an interface between the developing chick embryo and shell, allowing gas and calcium exchange. The stromal and epithelial components of the CAM provide an ECM similar to human epithelia rendering it suitable for the in vivo exploration of cell migration [16, 17].

Fertilized eggs were obtained from Henry Stewart, and Co. Ltd., allowed to settle overnight in a holding incubator at 19 °C then placed in a humidified incubator at 37 °C (Day 0). For live assays, eggs were windowed at 2–3 days by dropping the level of the albumen using a syringe and needle then making a small window in the eggshell. After 7 days of incubation, CAM invasion assays were conducted by seeding permanently transfected HT1080 or MDA-MB-231 cells expressing Green Fluorescent Protein (GFP) directly onto the CAM surface in a 1 mg/ml collagen solution (Rat tail Collagen Type 1, BD Bioscience). Eggs were harvested at different time points up to Day 14 of incubation. Live CAM images were taken using a Leica MZFLiii stereo microscope with DC500 camera and × 1 Leica lens with × 10 zoom, at room temperature. Harvested whole CAM was fixed and stained with Phalloidin Atto 565 (Sigma), DAPI (Sigma), α-rabbit Ki67 (abcam 16,667), α-GFP antibody: GFP rabbit IgG (A1112 Invitrogen) and secondary antibodies: Alexafluor 488 and Alexafluor 647 (Life Sciences). Tissue was embedded in OCT (Fisher) and sectioned using a Kryostat (Bright Model OTF).

### Decellularized CAM (dCAM)

CAM was decellularized using an adapted protocol based on that described by Medberry [18]. Briefly: CAM harvested at Day 9/10 of incubation was flash frozen in liquid nitrogen, thawed in ddH_2_O at 4 °C for 30 min, drained then stirred for 5 min at 37 °C in 0.02% trypsin/0.05% EDTA (Gibco, Sigma). Tissue was washed in ddH_2_O then exposed to the following reagents, with a ddH_2_O wash step between each: 3% TritonX-100 for 5-10 min, 1 M sucrose for 5 min, 4% deoxycholate (Sigma) for 5 min, 0.1% peracetic acid/4% ethanol (Sigma/Fisher) for 5–15 min, ddH_2_O for 5 min. dCAM was then freeze-dried. For cell culture, dCAM was exposed to UV radiation for 20 min then soaked for at least 24 h in PBS in a tissue culture incubator at 37 °C/5% CO_2_. PBS was replaced with DMEM/ 10% FBS/ 1% Pen/Strep and replaced in the incubator for 48 h. The medium was aspirated, and cells re-suspended at high density were seeded at low volumes, typically 0.5 ml at 1 × 10^5^, left to adhere (2–4 h) then additional medium added. Samples were prepared for mass spectrometry by solubilizing dCAM for 3 days according to the Medberry protocol. Centrifugation to pellet undissolved particles was conducted, and the pellet was re-suspended in DMSO. Both supernatant and DMSO were diluted in formic acid to a final concentration of 0.1%. Mass spectrometry was conducted by the Functional Genomics and Proteomics Facility at the University of Birmingham using ORBITRAP MS with CID fragmentation.

### Microscopy

A Nikon TiE fitted with a DS-Fi2 camera, Plan × 10/0.25 Ph1 DL and Plan Fluor EL WD × 20/0.45 Ph1 DM ∞/0.2 WD 7.4 lenses, an environmental chamber and a moveable platform stage (Prior Scientific) was used in conjunction with NIS Elements software for time-lapse microscopy. Image analysis was conducted using ImageJ MTrackJ plugin (ImageScience) and FIJI ImageJ software [19]. Live imaging of chicken embryo and CAM were obtained using a Leica MZFLiii stereo microscope with DC500 camera and × 1 Leica lens with × 10 zoom, at room temperature. Laser scanning confocal microscopy was conducted using either a Nikon A1 Plus or A1-R microscope at room temperature, using a × 20 Plan Apo VC × 20 DIC NR, NA 0.75 lens and × 60 1.40 Plan Apo ∞/0.17 WD 0.13, NA 1.4 lens. Images were acquired and prepared using NIS Elements, ImageJ and/or Photoshop CS6 Extended. Reflectance microscopy was conducted using a Leica TCS SP2 confocal microscope at room temperature with a Leica HCX PlanApo lbd.BL × 63 NA 1.4 oil immersion lens. Scanning Electron Microscopy (SEM) was conducted for gold sputter coated samples (Edwards S150b) using a Quanta FEI 600F.

A Zeiss Axio Vert.A1 epifluorescence microscope with an inverted lens and moveable platform was used to take individual images of live cells to monitor experiment progress and check for fluorescent protein expression. Lenses used were: × 5 Planar Plan Neofl Ph1 0.15 ∞ /0.17, × 10 Zeiss A Plan 0.25 Ph1 lens and a Zeiss LDA Plan × 20/0.35 Ph1 ∞ /1.0 (PS). A Leica DMi8 with DF33000G camera, moveable platform and onstage STR Tokai HIT incubator was used to take individual images of live cells using phase contrast microscopy with × 4/0.10 PH0 HI PLAN or × 10/0.25 PH1 N PLAN lenses.

### Statistics

A two-way ANOVA with Tukey’s test for multiple comparisons or a non-parametric test with Dunn’s test for multiple comparisons was conducted using GraphPad Prism 6, dependent on the data distribution.

## Results

### Cell morphology and migration speed differs with dimension

**Fig. 1:**
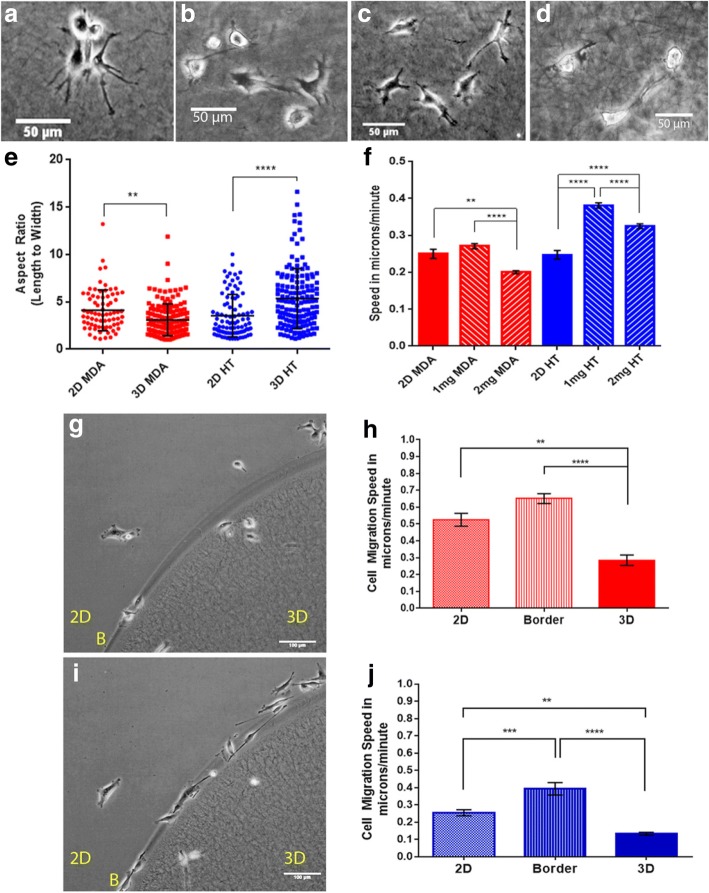
Cells adopt different morphologies and migration characteristics in 2D compared to 3D. MDA-MB-231 (MDA) cells adopted a more compact morphology when migrating in collagen (**b**) than over a collagen coated surface (**a**). However, HT1080 cells migrating in collagen were more elongated (**d**) than when migrating over it (**c**). Microscopy images were taken using a Nikon TiE phase contrast microscope and DS-Fi2 camera, a moveable stage and environmental chamber set at 37 °C with a continuous CO_2_/O_2_ supply. NIS Elements software was used for image capture and a Plan × 10/0.25 Ph1 DL lens; scale bars = 50 μm. The differences in aspect ratio are quantified in **e** where MDA-MB-231 and HT1080 cells are compared both to and in collagen (1 mg/ml). Cell migration speed is compared for cells moving over 2D collagen (2D) compared with cells moving in either 1 mg/ml or 2 mg/ml collagen for both MDA-MB-231 and HT1080 cells in **f**. Non-parametric Kruskal-Wallis Test with Dunn’s test for multiple comparisons was run for each condition. MDA-MB-231 and HT1080 cells were set up in a 2D/3D assay, and migration speed investigated in three different regions created: 2D, border, 3D. **g** and **h** show MDA cells behave differently according to their location and context as do HT1080 cells shown in **i** and **j** (2D, two dimensions, 3D, three-dimensional context, B, Border zone between the two contexts). Images show static shots taken from time-lapse movies, scale bars = 100 μm. Statistics were generated using a Two-way ANOVA with Tukey’s multiple comparisons tests using GraphPad Prism 6. Significance is shown: ** *p* ≤ 0.01, *** *p* ≤ 0.001, **** *p* ≤ 0.0001. n = 3

HT1080 fibrosarcoma cells and MDA-MB-231 breast cancer cells displayed different migratory characteristics when moving on as opposed to in a simple collagen-based context. When encapsulated in a collagen gel, MDA-MB-231 cells adopted a more compact morphology (Fig. 1a) than those migrating over the surface of a collagen gel (Fig. 1b). HT1080 cells, however, became less spread and more elongated when encapsulated within a collagen gel (Fig. 1c, d). Cell aspect ratio (cell length to width) was used to quantify these morphological differences which were found to be significant when compared with cells moving in 2D and 3D for each cell type, (Fig. 1e). Cell migration speed in 3D was significantly reduced for MDA-MB-231 cells as the collagen increased from 1 mg/ml to 2 mg/ml (mean values in μm/minute: 0.27, 0.20, difference 0.07, *n* = 3), Fig. 1f. For HT1080 cells the migration speed was significantly faster in the 3D matrix at 1 mg/ml (mean values in μm/minute: 0.25, 0.38, difference 0.13, *n* = 3) but reduced when the matrix density was increased from 1 mg/ml to 2 mg/ml (mean values in μm/minute: 0.38, 0.32, difference = 0.06). However, there was a significant difference in migration speed between 2D and 3D conditions for both collagen concentrations (Fig. 1f). A cell migration assay which provided both 2D and 3D environments for cells to move on, over or into, allowed cell migration to be tracked and analysed as cells moved within and between different environments. For both MDA-MB-231 (Fig. 1g, h) and HT1080 cells (Fig. 1i, j) cell migration was slower when cells moved across and/or into the 3D matrix (MDA-MB-231 mean values in μm/minute: 2D, 0.53, Border, 0.65, 3D 0.29 and for HT1080: 0.25, 0.39 and 0.13 μm/minute respectively, *n* = 3). At border zones, however, cells moved at greater speed migrating up and down the borders and appearing to use them as 1D migration tracks as well as transition zones. The results from these in vitro assays demonstrated the need for the cellular response to its surrounding environment to be considered when studying cell characteristics and behaviour.

### A stiffer more biologically relevant multi-dimensional context

**Fig. 2:**
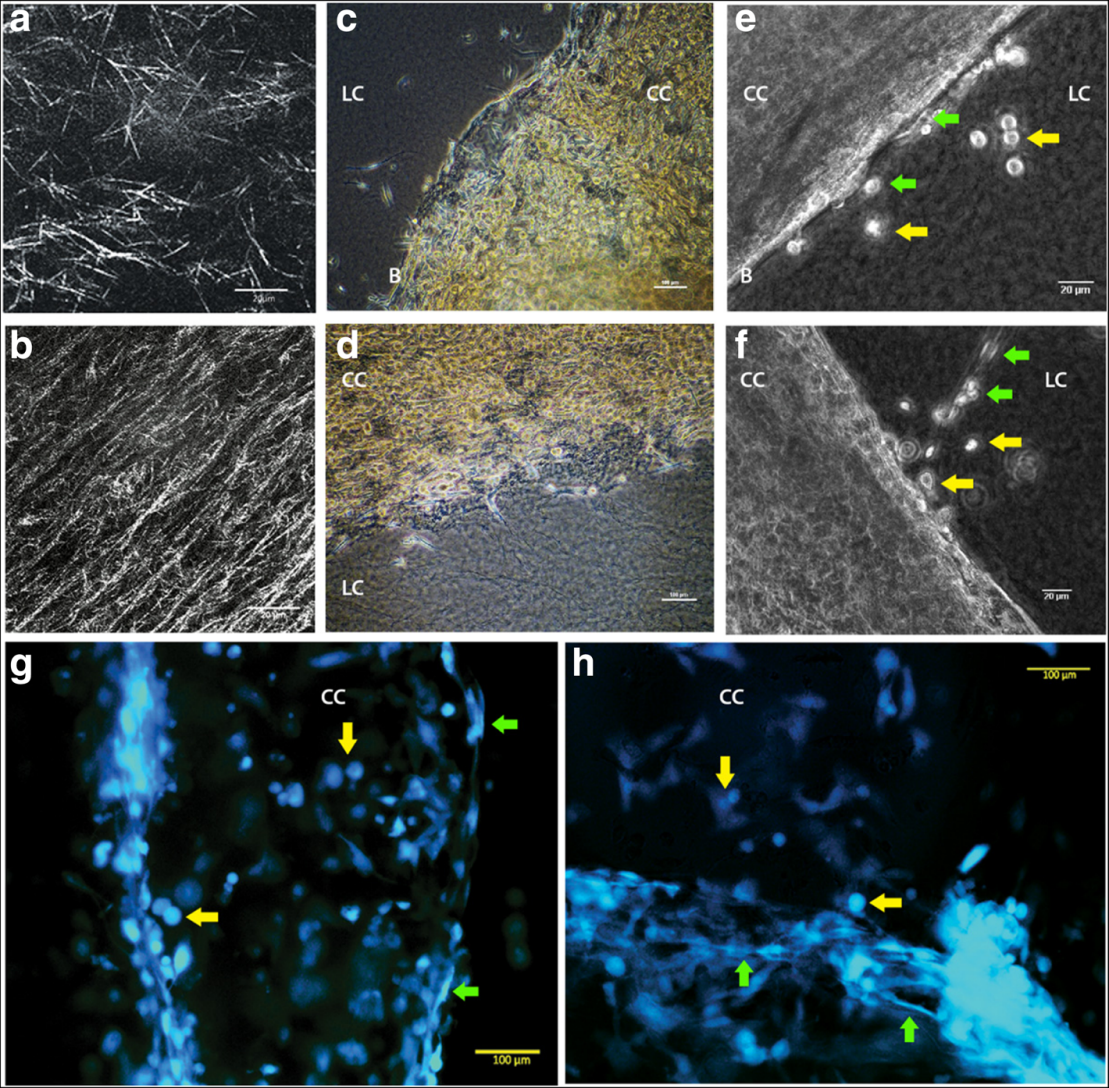
Compressed Collagen Assay provides a stiffer, more structured growth environment. A compressed collagen assay provides a stiffer, more structured growth environment for cell culture facilitating a greater range of cell morphology in colonizing cells. **a** and **b** show the collagen matrix in more detail via reflectance microscopy, **a** showing uncompressed collagen at 2 mg/ml and **b** compressed collagen derived from 2 mg/ml gel. (Image was taken using Leica TCS SP2 using 488 argon laser and Leica HCX PlanApo lbd.BL × 63 NA 1.4 oil immersion lens, at room temperature, scale bars = 20 μm). Cells seeded on compressed collagen (CC) set into a non-compressed collagen matrix (LC) colonized the compressed collagen in preference to migrating away from it. **c** and **d** show MDA-MB-231 cells colonizing the compressed collagen set into a lower density 1 mg/ml collagen gel. Few MDA-MB-231 cells emerged at the border zones (B) (scale bars = 100 μm). **e** and **f** show a few cells with a mainly rounded morphology which have escaped (yellow arrows) and moved away from the densely colonized compressed collagen into the lower density collagen (scale bars = 20 μm). In **f** emerging cells followed the contours of the compressed collagen disk (green arrows) while in **e** they appeared to form chains moving away from it (green arrows). Images **b**, **c**, **e**, **f**, were generated using a Nikon TiE phase contrast microscope, moveable stage, environmental chamber at 37 °C with a continuous CO_2_/O_2_ supply, DS-Fi2 camera, lenses: Plan × 10/0.25 Ph1 DL, Plan Fluor EL WD × 20/0.45 Ph1 DM ∞/0.2 WD 7.4. **g** and **h** show live GFP+ HT1080 cells colonizing the compressed collagen matrix. Cells had a diverse range of morphologies including rounded (yellow arrows) and elongated shapes (red arrows). Images were taken using a Zeiss Axio Vert.A1, DS-Fi2 camera, a Zeiss LDA Plan × 20/0.35 Ph1 ∞/1.0 (PS) lens at room temperature, scale bars = 100 μm

Using a compressed collagen technique developed for stem cell differentiation in biomaterials engineering [15], the 2D/3D assay was developed to provide a stiffer, more biologically relevant context for the study of cell invasion, migration and colonization. In setting a compressed collagen disc into a thin layer of uncompressed collagen, a multi-dimensional heterogeneous 3D environment was created with multiple border zones. Reflectance imaging of the collagen structure of the simple context (Fig. 2a) compared with the structural composition of the compressed collagen (Fig. 2b) showed that the compressed collagen comprised a denser network of aligned collagen fibres in comparison to the short more random arrangement of the uncompressed collagen context (Fig. 2a). Cells seeded onto the compressed collagen migrated over and colonized the stiffer matrix before migrating out onto the less dense collagen (Fig. 2c and d). Cells were also seen to move and transition with a range of morphologies, including elongated mesenchymal cell migration and spherical, compact morphologies (Fig. 2e, f). Cells appeared to move both individually (Fig. 2e, f) and collectively (Fig. 2f) within these contexts. Colonization of compressed collagen could be visualized more clearly using permanently transduced HT1080 GFP+ cells (Fig. 2g, h). The HT1080 GFP+ cells in the live images show that cells were able to adopt a range of morphologies from spherical to elongated on the compressed collagen.

The development of this more complex in vitro assay in which the entire range of cell morphologies adopted in vivo was observed, demonstrated the need for a stiffer more complex environment to support cell culture and the investigation of cell migration and colonization.

### CAM as an in vivo model for the study of cell migration and colonization

**Fig. 3:**
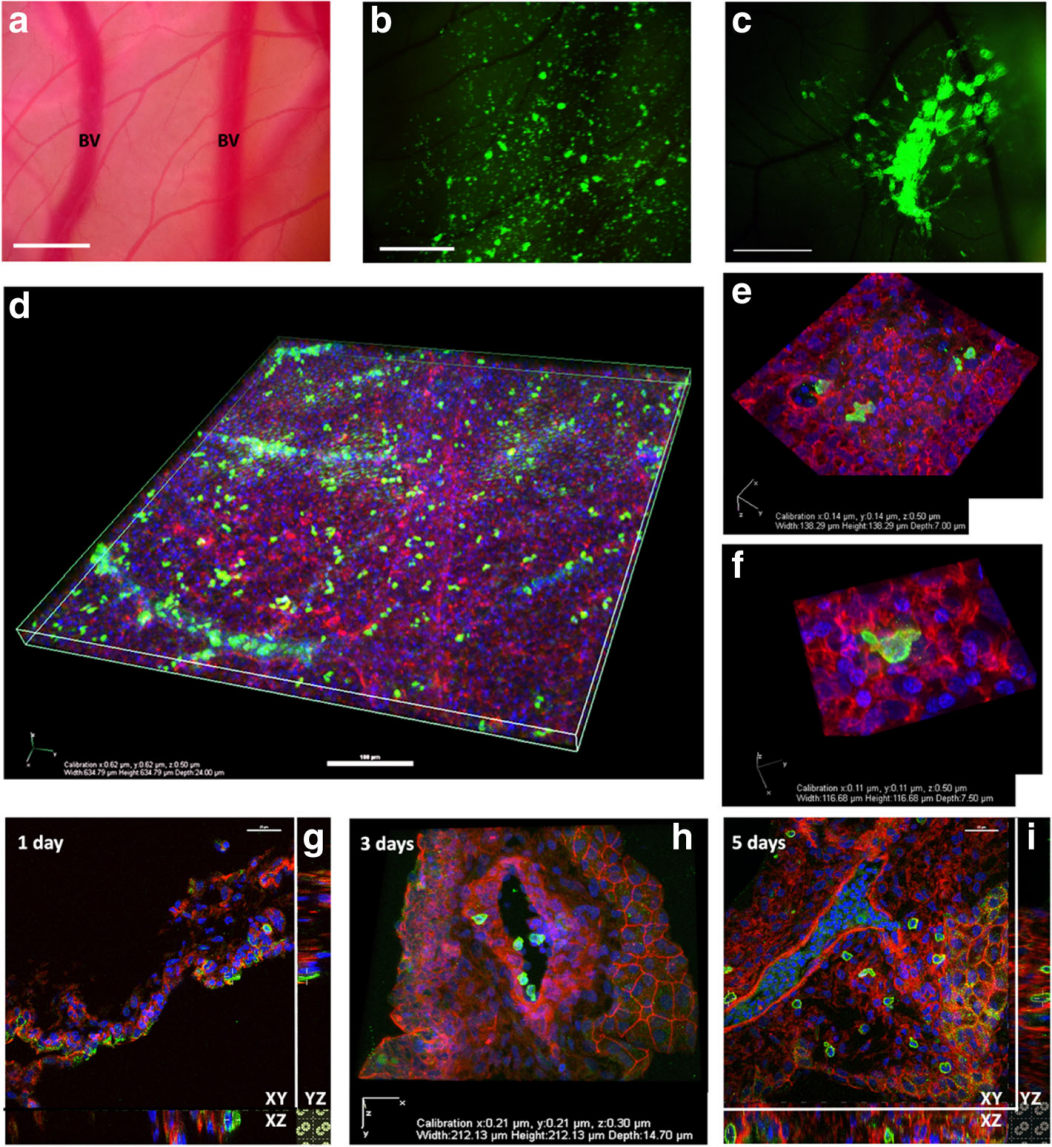
Cell migration and invasion can be explored by seeding GFP+ cells onto CAM. Cells re-suspended in 1 mg/ml collagen were seeded onto the surface of live CAM. **a**, bright field (BV = blood vessel) and **b**, **c**, epifluorescence images of live CAM with cells seeded over the surface (Leica MZFLiii stereo microscope with DC500 camera and × 1 Leica lens with × 10 zoom, at room temperature, scale bars = 1 mm). **d**, HT1080 GFP+ cells dispersed over fixed and stained whole CAM, scale bar = 100 μm. **e**, **f** Invaded HT1080 GFP+ cells show different morphologies in fixed and sectioned CAM. **g**, **h**, **i**: a timeline for the invasion of MCF7 GFP+ cells seeded onto CAM shows that cell invasion was evident 1 day after cell seeding and by day 5, cells could be seen within the vasculature and were well disseminated within CAM tissue (scale bars = 25 μm, image planes marked XY, XZ, YZ). Images D-I were taken using Nikon A1 plus confocal microscope, at room temperature. Image D was taken using a × 20 Plan Apo DIC N2, 0.75 NA lens. Image E-I were taken using a × 60 Plan Apo ∞/0.17 WD 0.13, NA 1.40 lens

The chicken egg chorioallantoic membrane (CAM) model, which has been well characterized and used in developmental biology and the investigation of angiogenesis, was explored as an in vivo model for the investigation of metastatic mechanisms, in particular, those of cell migration and colonization [16, 20–22]. Following a similar approach to the in vitro assays, GFP+ HT1080 or MDA-MB-231 cells suspended in a collagen gel were seeded directly onto the CAM surface. Outward migration of cells was observed at suitably chosen time points (Fig. 3a-c) and fixed, and stained CAM was probed to examine the extent of invasion and morphology of cells located within the CAM tissue (Fig. 3d-i). As the CAM is relatively thin (typically 30-100 μm), it was possible to use confocal microscopy to visualize and examine the spread of cells over and into intact CAM tissue as shown in Fig. 3d. Stained and sectioned CAM was used to examine the timescales of invasion (Fig. 3g-i) and the specifics of cell morphology and cell interaction with the surrounding tissue.

The complexity of the CAM tissue, however, limited the options for probing exogenous cell properties and interactions. The short developmental timescales of the chicken embryo model provided only a narrow window of opportunity for cell migration and colonization. However, if an acellular tissue structure could be developed offering the benefits of the complex ECM structure provided by the CAM without the complication of the chick cells, then this could be used as a platform for cell culture over longer time periods.

### Decellularized CAM (dCAM) as a 3D growth substrate

**Fig. 4:**
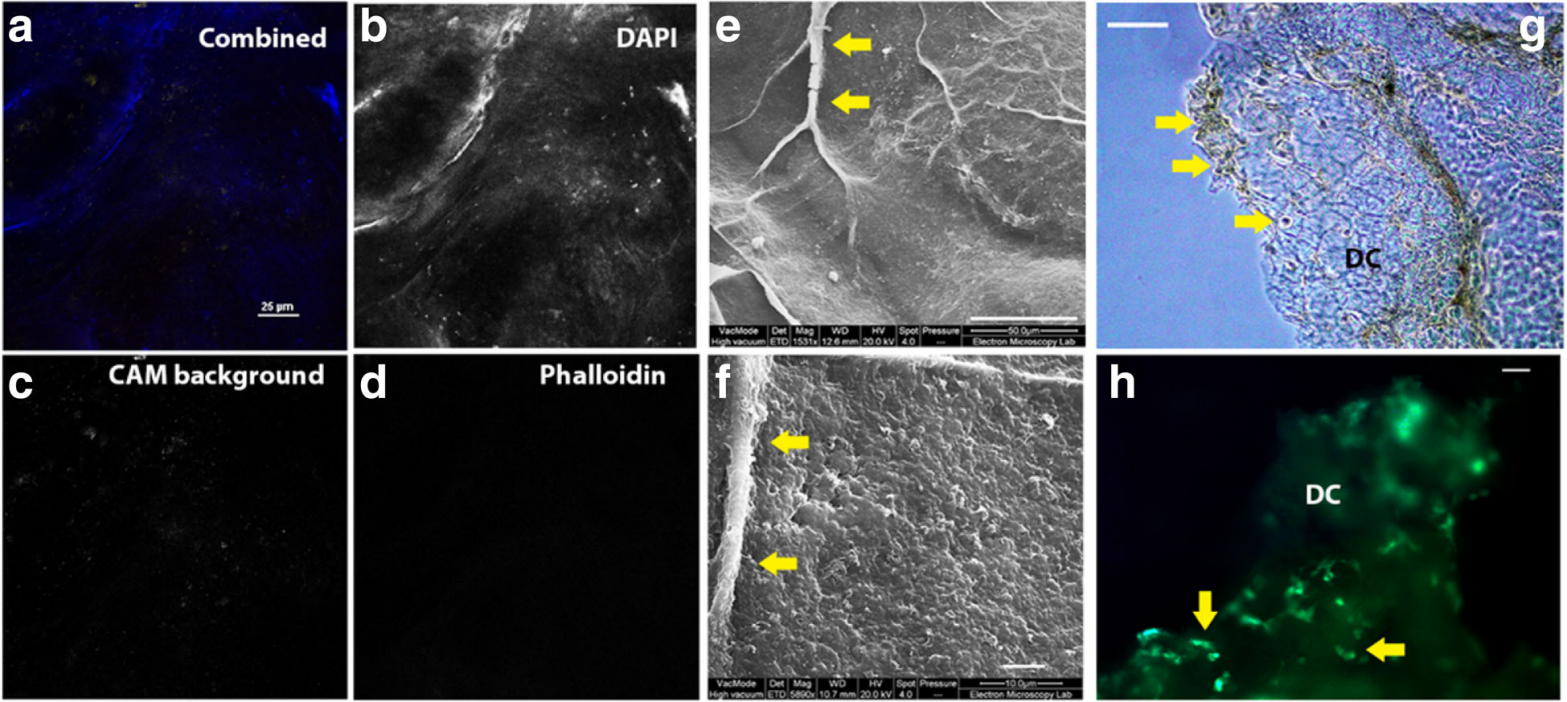
dCAM provides a collagen-rich 3D substrate for cell culture. Laser scanning confocal spectral unmixing was used to determine the residual components after decellularization of CAM (Nikon A1 Plus at room temperature using a × 60 1.40 Plan Apo ∞/0.17 WD 0.13, NA 1.4 lens). **a**, combined image, **b** DAPI only, **c** CAM background only, **d**, phalloidin for cellular actin cytoskeleton (scale bar for A = 20 μm). Scanning electron microscopy (SEM) was used to characterize the surface of the decellularized tissue (Quanta FEI), **e** and **f** show dCAM surface features including vasculature (yellow arrows) and fibrous extracellular matrix (scale bars: E = 50 μm, F = 5 μm). dCAM used as a growth matrix: **g** shows a bright field image of dCAM during colonization and **h** shows MDA-MB-231 GFP+ cells adhering and proliferating over the dCAM (DC, yellow arrows). Images G and H were taken using a Zeiss Axio Vert inverted epifluorescence microscope and × 5 Planar Plan Neofl Ph1 0.15 ∞ /0.17 lens with the DS-Fi2 camera, operating at room temperature, scale bars = 1 mm

CAM harvested from developing chicken embryos was decellularized and characterized to assess its suitability as an ECM based growth matrix for cell culture and the further exploration of cell migration and colonization. As the membranes are thin and delicate, careful optimization was necessary to ensure that minimal damage was caused while cells were removed. Phalloidin and DAPI staining used for whole CAM (Fig. 3d-i) showed that no remaining cell cytoskeleton material or nuclei remained following decellularization (Fig. 4a-d). Scanning electron microscopy enabled the decellularized CAM surface to be visualized in detail. The images showed that vasculature and acellular surfaces were preserved (Fig. 4e-f). Initial results for comparative mass spectrometry of CAM versus dCAM showed that foetal CAM proteins present in whole CAM had been removed during production of the decellularized CAM (Table 1 and Fig. 3). Used as a growth matrix, cells were seeded onto small sections of dCAM in tissue culture dishes and were observed to adhere and proliferate on the dCAM (Fig. 4g, h).

**Table 1:** Characterization of solubilized CAM and dCAM using mass spectrometry

	Id.	Description	Unique peptides	Protein Coverage
CAM				
1	P84407	Alpha-fetoprotein OS = *Gallus gallus* GN = AFP PE = 1 SV = 1 - [FETA_CHICK]	3	11.57
1	Q98UI9	Mucin-5B OS = Gallus gallus GN = MUC5B PE = 1 SV = 1 - [MUC5B_CHICK]	3	1.94
2	P01012	Ovalbumin OS = Gallus gallus GN=SERPINB14 PE = 1 SV = 2 - [OVAL_CHICK]	6	18.39
2	P02112	Hemoglobin subunit beta OS = Gallus gallus GN=HBB PE = 1 SV = 2 - [HBB_CHICK]	2	21.09
2	P00698	Lysozyme C OS = Gallus gallus GN = LYZ PE = 1 SV = 1 - [LYSC_CHICK]	3	33.33
2	O93532	Keratin, type II cytoskeletal cochleal OS = Gallus gallus PE = 2 SV = 1 - [K2CO_CHICK]	2	3.86
2	P01013	Ovalbumin-related protein X (Fragment) OS = Gallus gallus GN=SERPINB14C PE = 3 SV = 1 - [OVALX_CHICK]	2	19.4
dCAM				
1	Q90617	Lysosome-associated membrane glycoprotein 2 OS = Gallus gallus GN = LAMP2 PE = 2 SV = 1 - [LAMP2_CHICK]	2	6.12
1	P11722	Fibronectin (Fragments) OS = Gallus gallus GN=FN1 PE = 2 SV = 3 - [FINC_CHICK]	2	3.5
1	P02112	Hemoglobin subunit beta OS = Gallus gallus GN=HBB PE = 1 SV = 2 - [HBB_CHICK]	2	21.09
1	P02467	Collagen alpha-2(I) chain (Fragments) OS = Gallus gallus GN=COL1A2 PE = 1 SV = 2 - [CO1A2_CHICK]	2	1.62
2	P02112	Hemoglobin subunit beta OS = Gallus gallus GN=HBB PE = 1 SV = 2 - [HBB_CHICK]	2	21.09
2	P11722	Fibronectin (Fragments) OS = Gallus gallus GN=FN1 PE = 2 SV = 3 - [FINC_CHICK]	2	3.5

Key: 1, Supernatant; 2, Pellet

### dCAM as a 3D context for the investigation of cell behaviour

**Fig. 5:**
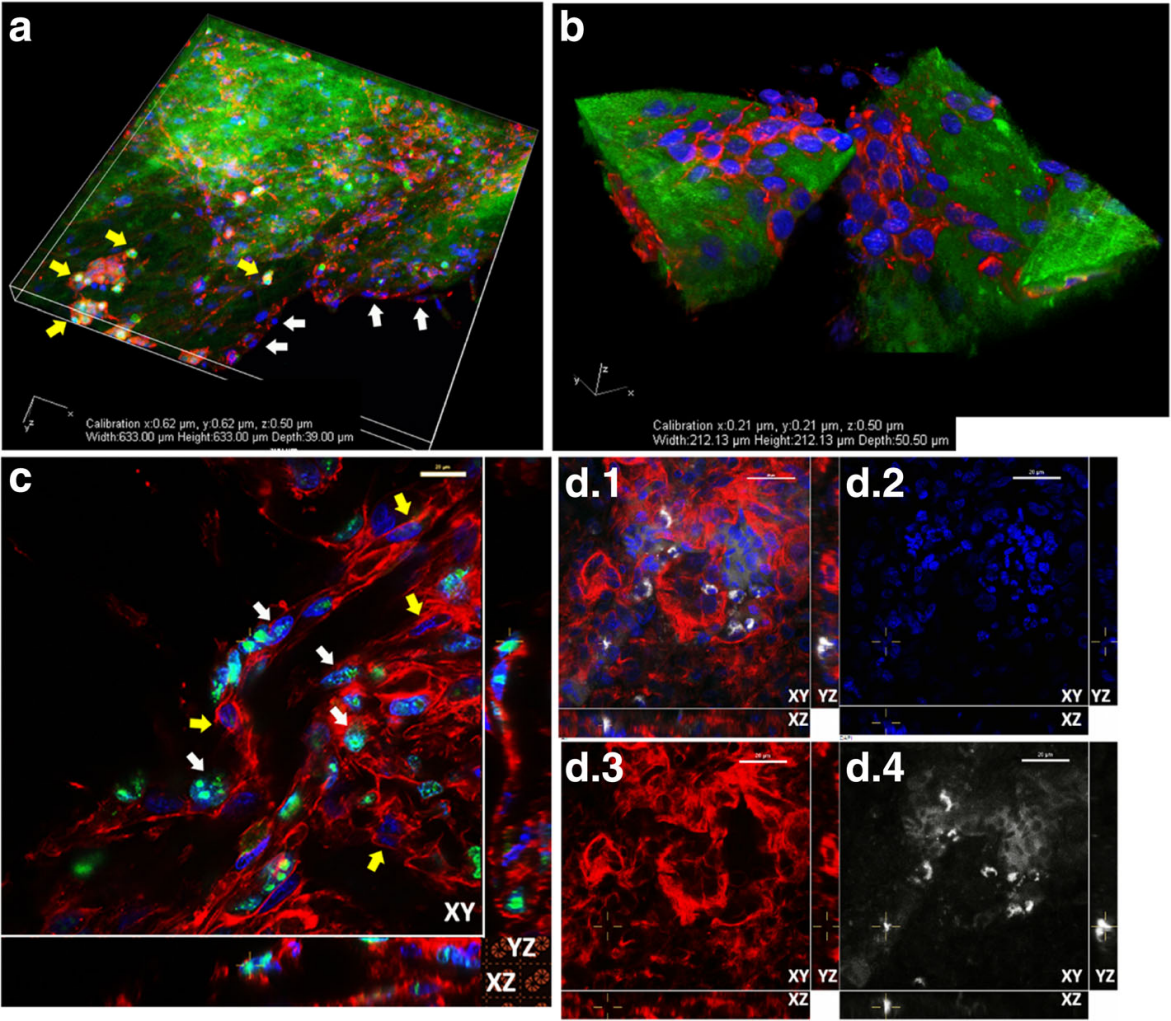
dCAM provides a structured 3D environment for studying cell proliferation and migration. **a**, dCAM partially populated with a co-culture of MDA-MB-231 (white arrows) and MCF7 GFP+ (yellow arrows) breast cancer cells stained with phalloidin for actin cytoskeleton (red) and DAPI nuclear stain (blue). **b**, MDA-MB-231 cells stained with phalloidin (red) and DAPI (blue) appear to have formed layers over the dCAM surface. **c**, Cells stained with cell proliferation marker Ki67 (Alexafluor 488, green), phalloidin (red), DAPI (blue) on dCAM. Differential Ki67 staining suggests that not all cells were actively proliferating (proliferating cells – white arrows, high Ki67, low Ki67 cells indicated with yellow arrows). **d.1**-**d.4**, Ki67 staining of human cells proliferating and migrating amongst chick CAM cells in invaded CAM (section): combined channels D.1, DAPI, blue (D.2); Phalloidin, red (D.3); Ki67/Alexafluor 647, white (D.4). Images were taken using Nikon A1R confocal microscope operating at room temperature, Image A using a × 20 Plan Apo VC DIC NR, NA 0.75 lens and Images B-D using a × 60 1.40 Plan Apo ∞/0.17 WD 0.13, NA 1.4 lens. Scale bars in C, D = 20 μm. Image planes for 3D images in C and D are marked XY, YZ, XZ

The decellularized CAM provided a simple and easy to use substrate upon which cancer cells could be seeded. Three different cell lines were used: MCF-7, MDA-MB-231 and HT1080 cells. These were seeded and allowed to proliferate as either a monoculture (Fig. 5b) or as a co-culture (Fig. 5a). Populated dCAM was fixed and stained, and 3D images obtained using regular confocal imaging without sectioning, allowing cell-cell and cell-matrix interactions to be visualized in intact tissue. Ki67 staining for cell proliferation in HT1080 cells cultured on dCAM (Fig. 5c) showed that cells were at different stages in the cell cycle while the dCAM was being colonized. Comparative Ki67 staining in seeded CAM (Fig. 5d) showed just a few human cells proliferating amongst the chick cells of the CAM.

### Testing the pipeline approach

**Fig. 6:**
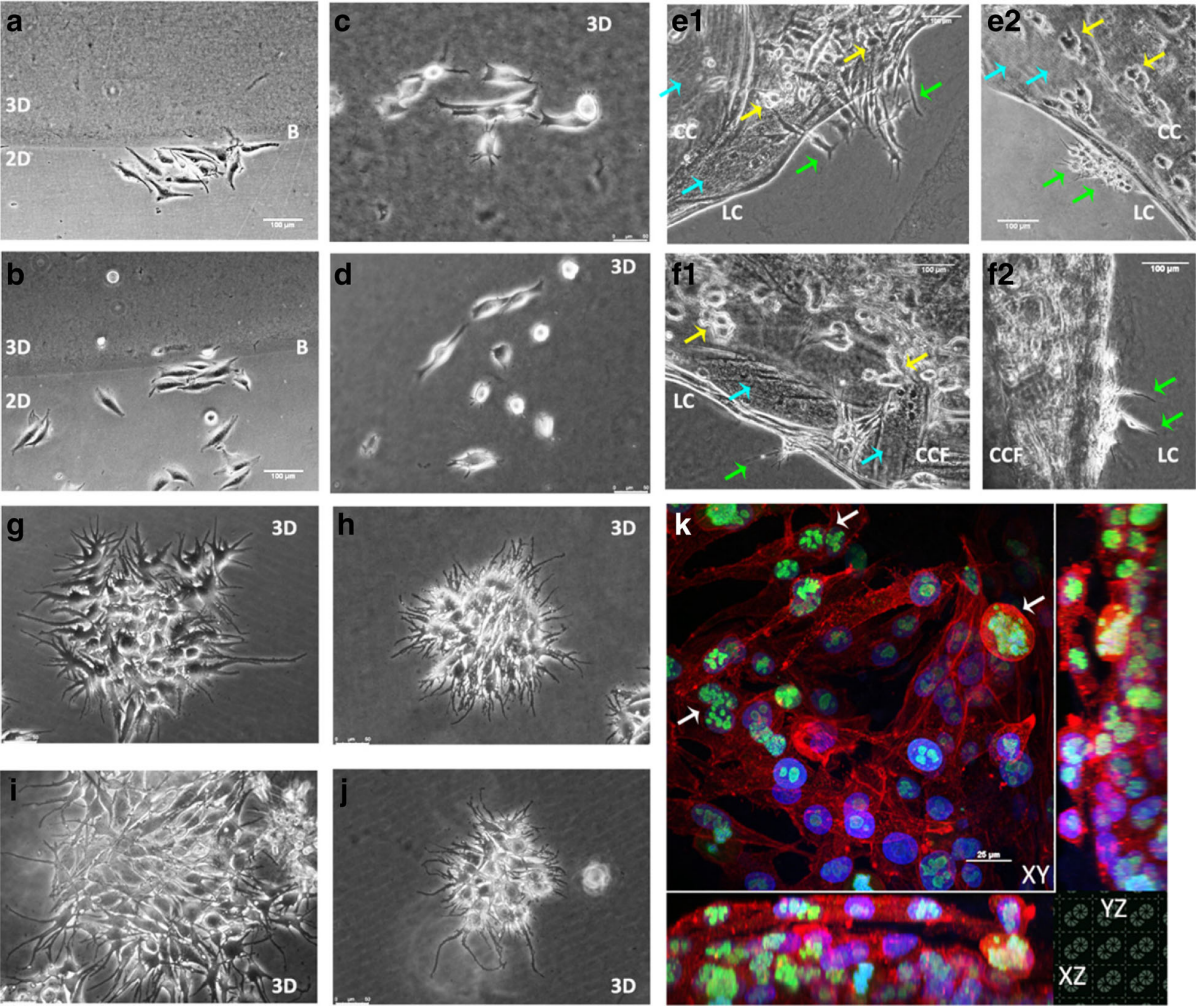
Using the pipeline to characterize the escape and colonization of SK-MEL-28 melanoma cells. Melanoma cells were introduced into four pipeline assays, providing the opportunity to compare and contrast cell behaviour in each context. In the 2D/3D assay, melanoma cells adopted an elongated morphology on 2D plastic, at border zones (white B) and on top of the 3D matrix. Collagen was used at two different concentrations: **a**, 1 mg/ml and **c**, 2 mg/ml and with 10 μg/ml fibronectin: **b**, 1 mg/ml collagen + fibronectin and **d**, 2 mg/ml collagen + fibronectin. When melanoma cells were encapsulated in collagen (**g**, 1 mg/ml, **h** 2 mg/ml) or collagen with 10 μg/ml fibronectin (**i**, 1 mg/ml + fibronectin, **j**, 2 mg/ml + fibronectin), they proliferated to form small tight colonies in the 2 mg/ml gels and looser spread structures at 1 mg/ml. In a 2 mg/ml compressed collagen matrix, **e1 - e2**, melanoma cells partially colonized the matrix (blue arrows show uncolonized matrix) before escaping into the surrounding lower density matrix (LC). Melanoma cells within the compressed collagen (CC) adopted ovoid groupings (yellow arrows) cells becoming elongated with narrow filopodia upon escape (green arrows). Similar behaviours were observed in compressed collagen with fibronectin (CCF), **f1 - f2**. Melanoma cells seeded onto dCAM, cultured for 10 days proliferated to form layers. Ki67 staining (green) indicated that cells were actively dividing within each layer – white arrows. Phalloidin – red. Dapi – blue. Images **a**, **b**, **e1**–**2**, **f1**–**2** were generated using Nikon TiE timelapse system and Plan × 10/0,25 Ph1 DL lens and NIS Nikon Elements software. Scale bars = 100 μm. Images **c**, **d**, **g**-**j** were generated using a Leica DMi8 inverted microscope, Leica DF33000G camera, Tokai Hit STR stage top incubator, ND × 4/0.10 PH0 HI PLAN and × 10/0.25 PH1 N PLAN achromatic objective lenses. Scale bars = 50 μm. Image **k** was generated using Nikon A1-R confocal microscope with a × 60 1.40 Plan Apo ∞/0.17 WD 0.13, NA 1.4 lens. Scale bar = 25 μm

SK-MEL-28 melanoma cells were introduced into each of the in vitro assays of the proposed pipeline: 2D/3D assay, 3D encapsulation in either collagen or collagen supplemented with fibronectin, Compressed Collagen (CC) or Compressed Collagen with fibronectin (CCF) and dCAM (Fig. 6). The melanoma cells adopted an elongated morphology at border zones, on collagen (Fig. 6a, b) or on collagen supplemented with fibronectin (Fig. 6c, d) used at two different concentrations (1 mg/ml and 2 mg/ml collagen). However, when encapsulated within collagen gels or collagen gels supplemented with fibronectin, cell colonies within the denser matrix (Fig. 6i, j) showed a more compact arrangement compared with those observed in the lower density matrix (Fig. 6g, h). SK-MEL-28 cells encapsulated in compressed collagen or compressed collagen supplemented with fibronectin were seen to partially populate the stiffened collagen matrix before invading into the surrounding lower density collagen matrix. Cells escaping the compressed collagen adopted an elongated morphology with filopodia extending out into the lower density gel matrix (Fig. 6e, f). When colonizing the decellularized chorioallantoic membrane (dCAM), melanoma cells formed multiple layers, quite unlike the behaviour displayed when they were cultured on 2D. Ki67 staining indicated that the melanoma cells were actively dividing within each of the layers observed.

## Discussion

Recent studies focussing on the progression of cancer have highlighted the importance of the tumour microenvironment in both preventing and facilitating the outward spread of cancer [7, 23, 24]. In vitro models of metastasis have typically been simple and have lacked the complexity and structure of the tissue environment, whereas in vivo models have been expensive, difficult to set up and limited in their application. While any model has inherent limitations, a complex 3D tissue culture model representative of the appropriate native environment which can be manipulated and controlled under experimental conditions would provide a good platform for the study of cellular and molecular mechanisms [11, 25].

**Fig. 7:**
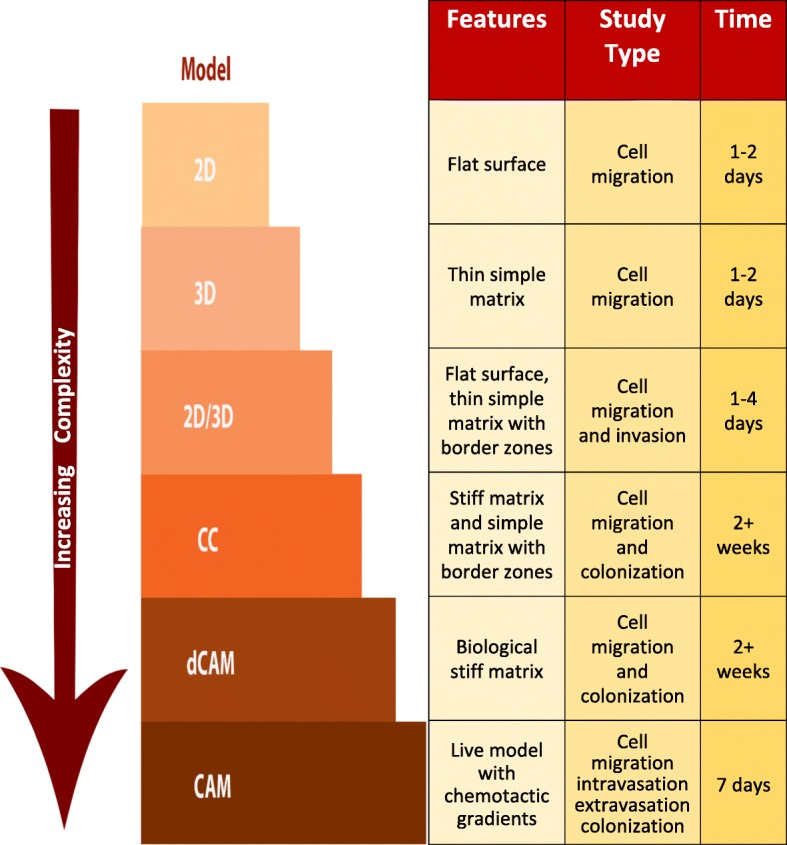
A Pipeline Approach to the Experimental Modelling of Metastasis. The models discussed and presented in this paper are shown in order of increasing complexity: 2D, two-dimensional cell migration assay; 3D, three-dimensional cell migration assay; 2D/3D assay; CC, compressed collagen assay; dCAM, decellularized chorioallantoic membrane assay; CAM, chorioallantoic membrane assay. The structural features are listed along with the type of study that the model is suitable for and the time frame for its use

There has been much emphasis on the extracellular matrix as a transitory layer and essential conduit for migrating cells, as well as being a contributor to the tumour microenvironment [8, 26, 27]. Extracellular matrix materials have therefore proved a popular starting point for much of the recent research in this area. Using collagen, the main constituent of ECM as a starting point, we have developed a set of assays which build on the existing assays used in the field to provide a pipeline for the comparison and contrast of cell behaviours in increasingly complex ECM based 3D environments. This pipeline approach is illustrated in the model shown in Fig. 7. In the 2D/3D assay, the simplest of the pipeline assays, a range of cell behaviours and morphologies could be observed and quantified at different contextual locations within the same assay. This combines conventional approaches to cell migration in which cell behaviours can be observed in both 2D and 3D and additionally introduces a border zone at which cell transition between 2D and 3D contexts can be observed. Pleomorphic cell behaviour observed in this assay demonstrated the adaptability of cells to a simple context with only limited variability in surface and constituents. The second and more complex assay described here, the compressed collagen assay (CC), provided a stiffer and more elastic context for cell study, with the added benefit of multiple regions: the stiff compressed collagen, two different border zones, a simpler collagen matrix and a two-dimensional planar surface. Colonization within this assay took place over a much longer period than was possible in either a simple collagen context or a 2D monolayer. It was possible to observe and quantify both cell morphology and migration behaviours in this more complex environment, one which not only facilitated the extended observation of cell-cell and cell-ECM interactions but enabled a variety of cell behaviours to emerge. In this context, the cells adopted a range of morphologies more closely resembling those seen in vivo. Following the recent development of a thin high-density fibrillary collagen layer for the study of proteolytic invasion [28] the compressed collagen assay developed here provides an alternative dense collagen environment which could be used to further explore invasive and migratory behaviour in a flexible manner. These collagen-based assays were further augmented with fibronectin when testing the pipeline, to demonstrate that additional ECM constituents could be introduced to further explore cell behaviours.

The chick-derived decellularized ECM (dCAM), provided a still more complex 3D context with the natural features and variation of an in vivo environment. Seeded cells were able to divide and colonize the ECM environment over a longer time period, extending the potential culture time to weeks rather than days. Co-culture was also supported with cell types being introduced at different time points within the tissue culture program, and cell-cell interactions and contribution within the 3D environment observed. Variability in staining for the proliferation marker Ki67 suggested that while some cells were actively proliferating, others may have become quiescent indicating that cells may be able to differentiate and settle in the dCAM environment, as opposed to the continuous cell division typically observed during cell culture on 2D surfaces. Behaviours seen within the live CAM model were explored further within dCAM providing the opportunity to move between a complex live model and a structured biologically relevant culture substrate in a controlled in vitro environment. A significant advantage of using the decellularized tissue as a substrate for the study of cell interactions and behaviour in 3D was that imaging could be conducted of cells in situ, either live or fixed but without sectioning, thus allowing populated tissue to remain intact and therefore minimising the introduction of artefacts. In this way cell morphology in both CAM and dCAM could be identified and quantified in a similar way to that carried out in the simple 2D/3D assay. Finally, the introduction of melanoma cells into each of the in vitro assays of the pipeline demonstrated the differential response of cells to each environment in this context based approach.

We feel this pipeline approach provides a robust set of assays which can be used to explore the escape, invasion and colonization steps of metastasis. During assay development we have introduced a variety of cancer cell types including cells from a primary sarcoma (HT1080), breast cancer cells which are oestrogen positive and considered non-invasive (MCF-7) and triple-negative breast cancer cells which are metastatic and highly invasive (MDA-MB-231). We have further tested the pipeline with melanoma cells originating from skin carcinoma (SK-MEL-28).

## Conclusions

Taken together these ECM-based assays provide an opportunity to study cell interactions and behaviours in contexts of increasing complexity, with the ability to observe, record and quantify cell behaviours and events while preserving the structural integrity of each cultured tissue environment. The inherent flexibility of the models provides the opportunity for the manipulation of experimental conditions across and within the assays so that cell behaviours in each context can be compared and contrasted under different experimental conditions. Thus a pipeline approach can be adopted for the elucidation of cell migration and colonization, important steps in the metastatic process. The testing of drug compounds would also benefit from such a pipeline approach employing a range of versatile tissue culture tools to explore and elucidate the mechanisms and effects of drug interactions in increasingly complex 3D contexts which can be manipulated and then reproducibly replicated.

## Abbreviations

1D: One-dimensional
2D: Two-dimensional
3D: Three-dimensional
CAM: Chick chorioallantoic membrane
CC: Compressed collagen
CCF: Compressed collagen with fibronectin
dCAM: Decellularized chick chorioallantoic membrane
ECM: Extracellular matrix
SEM: Scanning electron microscopy
